# Acceptability of cognitive remediation for schizophrenia: a systematic review and meta-analysis of randomized controlled trials

**DOI:** 10.1017/S0033291722000319

**Published:** 2023-06

**Authors:** Antonio Vita, Stefano Barlati, Anna Ceraso, Giacomo Deste, Gabriele Nibbio, Til Wykes

**Affiliations:** 1 Department of Clinical and Experimental Sciences, University of Brescia, Brescia, Italy; 2 Department of Mental Health and Addiction Services, ASST Spedali Civili of Brescia, Brescia, Italy; 3 Department of Psychology, Institute of Psychiatry, Psychology and Neuroscience, King's College London, London, UK; 4 South London and Maudsley NHS Foundation Trust, Maudsley Hospital, Denmark Hill, London, UK

**Keywords:** Acceptability, cognitive remediation, drop-out, meta-analysis, schizophrenia, systematic review

## Abstract

**Background:**

Acceptability is an important factor for predicting intervention use and potential treatment outcomes in psychosocial interventions. Cognitive remediation (CR) improves cognition and functioning in people with a diagnosis of schizophrenia, but its acceptability, and the impact of participants and treatment characteristics, remain to be investigated. Few studies provide a direct measure of acceptability, but treatment drop-out rates are often available and represent a valid surrogate.

**Method:**

The systematic search conducted for the most comprehensive CR outcomes database for schizophrenia was updated in December 2020. Eligible studies were randomized clinical trials comparing CR with any other control condition in patients diagnosed with schizophrenia spectrum disorders and that also reported drop-out in treatment and control arms separately. Acceptability was measured as odd-ratios (OR) of drop-out.

**Results:**

Of 2119 identified reports, 151 studies, reporting 169 comparisons between CR and control interventions with 10 477 participants were included in the analyses. The overall rate of drop-out was 16.58% for CR programs and 15.21% for control conditions. In the meta-analysis, no difference emerged between CR interventions and controls [OR 1.10, 95% confidence interval (CI) 0.96–1.25, *p* = 0.177]. Factors improving acceptability were: inpatient only recruitment, participants with fewer years of education and lower premorbid IQ, the presence of all CR core elements, and the presence of techniques to transfer cognitive gains into real-world functioning.

**Conclusions:**

CR for people diagnosed with schizophrenia is effective and has a good acceptability profile, similar to that of other evidence-based psychosocial interventions.

## Introduction

### Background

Cognitive remediation (CR) for people diagnosed with schizophrenia is a behavioral training-based intervention targeting cognitive processes (Wykes, Huddy, Cellard, McGurk, & Czobor, 2011). It produces significant gains not only in cognitive performance, but also in real-world psychosocial functioning, as demonstrated in systematic and recent meta-analyses (Kambeitz-Ilankovic et al., 2019; Lejeune, Northrop, & Kurtz, 2021; Vita et al., 2021), even for people who are more clinically compromised (Vita et al., 2021). So, there is little doubt that CR is an important asset in aiding people living with schizophrenia to achieve their recovery goals.

CR implementation is not widespread despite recent guidance (Keepers et al., 2020; National Institute for Health and Care Excellence, 2020), and there is still some reluctance to include it in everyday clinical practice (Vita & Barlati, 2019; Wykes, 2018). Acceptability is a key factor in real-world clinical settings as it is a predictor of intervention use and potential outcomes under real-world conditions. We know from the most recent meta-analysis that longer treatments produce better outcomes (Vita et al., 2021), so factors fostering participation as well as potential barriers to treatment delivery are important to discover. Acceptability is a complex construct, which can be assessed in different ways, depending on the theoretical framework in which it is evaluated (Carter, 2007). In healthcare interventions acceptability can be estimated through patient feedback, or by treatment drop-out rates (Sekhon, Cartwright, & Francis, 2017).

In the few studies that have explored acceptability directly, patient views of CR interventions were generally positive (Bryce, Warren, Ponsford, Rossell, & Lee, 2018; Contreras, Lee, Tan, Castle, & Rossell, 2016; Nemoto et al., 2021; Rose et al., 2008), and participants' satisfaction and judgements of CR effects matched their efficacy data (Rose et al., 2008). In the absence of direct measures, acceptability needs to be investigated indirectly by considering treatment drop-out. For CR studies this is the reported levels of drop-out by trial treatment arm. Meta-analyses have found trial attrition rates of 13–14% for non-pharmacological interventions, though with considerable between-study heterogeneity (Szymczynska, Walsh, Greenberg, & Priebe, 2017; Villeneuve, Potvin, Lesage, & Nicole, 2010). CR randomized trials and naturalistic studies, including those with large samples, report drop-out rates of 10–17%, suggesting that it has comparable acceptability (Horan et al., 2018; Mueller, Schmidt, & Roder, 2015; Østergaard Christensen et al., 2014; Soumet-Leman, Medalia, & Erlich, 2018; Tan et al., 2019), while other large trials found much higher drop-out rates (Donohoe et al., 2018; Kantrowitz et al., 2016; Mahncke et al., 2019; van Oosterhout et al., 2014).

It is unclear which variables influence attrition in CR studies, and some suggested are the design, setting, participant socio-demographic and clinical characteristics, treatment duration, and specific treatment components (Best et al., 2020; Fiszdon, Kurtz, Choi, Bell, & Martino, 2016; Saperstein, Lynch, Qian, & Medalia, 2020; Sedgwick, Hardy, Newbery, & Cella, 2021).

The core ingredients of CR (presence of an active and trained therapist, repeated practice of cognitive exercises, structured development of cognitive strategies, and use of techniques to improve the transfer of cognitive gains to the real world) identified in a recent expert consensus (Bowie et al., 2019) influence treatment effectiveness (Vita et al., 2021). We do not know if these same components have an impact on acceptability nor whether any patient-related characteristics could identify candidates who would complete treatment and therefore be more likely to receive a treatment benefit.

### Aims of the study

To investigate CR acceptability (trial drop-out rate) between treatment and control arms in randomized clinical trials and assess if differences were moderated by patient characteristics or beneficial treatment components.

## Methods

The review was conducted in accordance with the Preferred Reported Items for Systematic Review and Meta-analysis (PRISMA) guidelines (Moher, Liberati, Tetzlaff, Altman, & Group, 2009), and can be viewed as a complementary analysis to Vita et al. (2021). The literature search was completed on Dec 23, 2020, and comprised a systematic search of 3 electronic databases (PubMed, Scopus and PsycInfo) using the terms [(‘cognitive’ or ‘cognit*’) AND (‘training’ or ‘remediation’ or ‘rehabilitation’ or ‘enhancement’) AND (‘schizophrenia’ or ‘psychosis’) AND (‘random*’ or ‘randomized control trial’ or ‘clinical trial’)], a manual search of Google Scholar with combinations of the same keywords, and manual inspection of reference lists of emerging reviews and included papers. Studies previously excluded due to the absence of outcomes of interest or not providing usable outcome data were re-assessed for eligibility.

Eligible studies were randomized trials recruiting participants with a diagnosis of schizophrenia spectrum disorders (at least 70% of the total sample), comparing CR interventions fulfilling the standard Experts Workshop definition (2010) to any control condition other than CR. For this meta-analysis we chose studies explicitly reporting drop-out in each treatment arm. CR could be either delivered as a stand-alone treatment or integrated with other adequately controlled psychosocial interventions. No restriction was applied either to the treatment setting or the duration and delivery mode of CR interventions. The screening was conducted by 2 independent reviewers with disagreements resolved by a third author. Only articles published in English in peer-reviewed journals were considered. The methodological rigor of included studies was evaluated using the Clinical Trials Assessment Measure (CTAM) (Tarrier & Wykes, 2004) by at least 2 reviewers, who also extracted data independently.

### Outcomes of interest

The main outcome was the reported number of trial drop-outs due to any reason in each treatment arm. Drop-outs were individuals providing consent to participate in the study, randomized to one of the treatment arms and leaving the study before the end of the active treatment phase. Drop-out occurring after treatment was not considered. All drop-out definitions were considered valid, with no threshold for the minimum number of attended treatment sessions. If the reasons for drop-out were reported then this information was also extracted. A separate analysis was conducted including only drop-out due to lack of motivation, i.e., participants who withdrew consent to participate during the active treatment period or who explicitly considered the treatment tedious or too intensive.

### Outcome measures

Acceptability was measured as odd-ratios (OR) of drop-out, with 95% confidence interval (CI), calculated according to the following formula: OR = (drop-out from CR interventions/completers of CR interventions)/(drop-out from control interventions/completers of control interventions). For studies with multiple treatment arms, each eligible comparison between CR interventions and control conditions was considered separately. As including multiple comparisons within a study could lead to a potential bias of dependent effects, sensitivity analyses were conducted introducing only one comparison per study (Rücker, Cates, & Schwarzer, 2017; Van den Noortgate, López-López, Marín-Martínez, & Sánchez-Meca, 2013).

### Meta-analytic procedures

Given the expected high level of participant clinical heterogeneity of included studies, a random-effect approach was preferred. A sensitivity analysis was also performed using a fixed-effect approach. Statistical heterogeneity was investigated through visual inspection of forest plots and assessment of Q-test and I^2^ statistic. All meta-analyses were performed using Comprehensive Meta-Analysis version 3.0 (Biostat, Englewood, NJ, USA, 2013). Descriptive statistics and analyses were performed using SPSS 22 (SPSS Inc., Chicago, IL, USA, 2005). *p* values <0.05 (2-tailed) were considered significant for all analyses.

### Moderator effects

Subgroup, sensitivity, and meta-regression analyses were conducted to explore the effect of study methods, participants, and interventions characteristics on acceptability as identified or suggested by other investigators. We also carried out a sensitivity analysis including only trials conducted in real-world settings, and subgroup analysis to compare pilot/feasibility studies to more rigorous trials. The list of moderator variables explored is available in Appendix 1.

### Certainty of the evidence

The risk of publication bias was assessed by visual inspection of funnel plots and Egger test for asymmetry (Egger, Smith, Schneider, & Minder, 1997). Other determinants of the quality of evidence such as consistency, precision, and directness were explored using experts' recommendation (Schünemann, 2013).

## Results

**Fig. 1.**
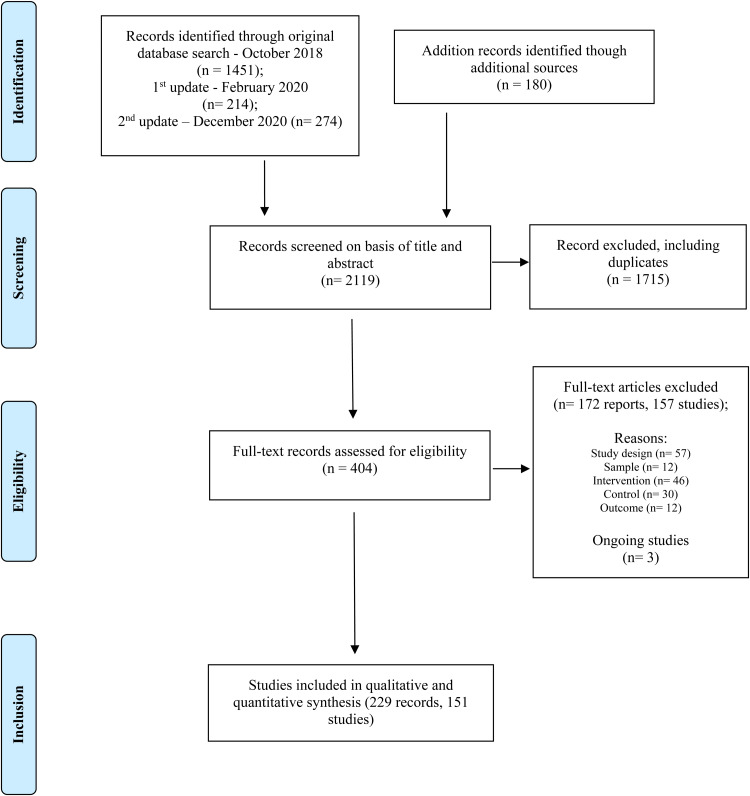
PRISMA flow diagram.

The study selection procedure is shown in Fig. 1. A total of 151 studies, reporting 169 comparisons between CR and control interventions with 10 477 participants were included in the analyses; 3 ongoing studies were also identified (see Appendix 2). Included participants were representative of people living with schizophrenia and using mental health services, from individuals experiencing first episodes to those with thirty-year illness duration, and at different stages of recovery.

The mean CR treatment duration was 15.82 ± 15.45 (range 2–104) weeks; the four core ingredients of CR (Bowie et al., 2019) were well represented (single elements present in ⩾70% cases); however, only half (66, 48.88%) included all of them. CR was delivered in the context of a structured psychiatric rehabilitation program in 48 (28.9%) cases.

### Included studies

Sixty-five studies were conducted in Europe, 41 in the US, 27 in Asia, 7 in Middle East countries, 5 in Canada, 4 in Australia and 2 in Brazil. Eighty-three studies recruited outpatients, 50 included inpatients from acute and residential care services, and 18 recruited from both. Most took place in real-world clinical practice sites (101, 66.9%), while some trials were conducted in specific settings (e.g. 20 research-oriented sites, 6 home-based or low-support programs, 4 forensic settings, 1 supported university program). Thirty-nine (25.8%) studies were explicitly defined as pilot/feasibility trials. Descriptive data are reported in detail in Table 1.

**Table 1.** Descriptive characteristics of 151 included studies reporting data on 169 intervention-control comparisons

Variable	Studies *N*	Mean ± s.d./*n* (%)	Range
Study characteristics			
Design	151		
Single-center trial		102 (67.5%)	
Multicenter trial		49 (32.5%)	
Setting	151		
Outpatients		83 (55.0%)	
Inpatients		50 (33.1%)	
Both		18 (11.9%)	
Sample size (randomized participants)	151	69.38 ± 44,40	10–34
Methodological quality	151		
Total CTAM score (max 100)		63.17 ± 14.10	26–92
Trials with ⩾65 points		80 (53%)	
Trials with <65 points		71 (47%)	
Drop-out rate (%)	151	13.34 ± 12.26	0–49.06
Cognitive remediation		14.37 ± 13.82	0–58.06
Control condition		12.68 ± 12.01	0–52.94
Including only participants diagnosed with schizophrenia	151	69 (45.7%)	
Providing payment to included participants	151	28 (18.5%)	
Conducted in clinical practice site	151	101 (66.9%)	
Pilot or feasibility trials	151	39 (25.8%)	
Comparison category	169		
TAU		61 (36.1%)	
Active TAU		24 (14.2%)	
Nonspecific active control		50 (29.6%)	
Evidence-based control		34 (20.1%)	
Participant characteristics			
Age (years)	150	36.82 ± 7.40	15.29–65.25
Gender (% females)	143	32.38 ± 13.63	0–75
Education (years)	105	11.88 ± 1.38	7.69–17.05
Premorbid IQ	62	96.38 ± 7.87	74.84–111.40
Age of onset (years)	98	23.30 ± 2.52	13.43–28.80
Duration of illness (years)	99	13.83 ± 6.21	0.67–29.67
Baseline therapy dose (CPZeq)	72	557.76 ± 284.45	182.5–1609.66
Baseline symptoms severity (PANSS score)	91	68.33 ± 15.87	41.89–118.40
Baseline severity category (CGI categories)	91		
Mild		40 (44.0%)	
Moderate		31 (34.1%)	
Marked		14 (15.4%)	
Severe		6 (6.6%)	
Treatment characteristics (*N* = 169 comparisons)			
Duration (weeks)	166	15.83 ± 15.45	2–104
Intensity	157		
Sessions/week	157	2.56 ± 1.28	0.5–7.8
Hours/week	152	2.48 ± 1.41	0.42–10
Format of delivery	166		
Individual sessions		74 (44.6%)	
Group sessions		71 (42.8%)	
Both		21 (12.7%)	
Method of delivery	166		
Computer-assisted		72 (43.4%)	
Pencil-and-paper		50 (31.1%)	
Both		44 (26.5%)	
Core elements included	166		
1. Active and trained therapist		133 (89.1%)	
2. Repeated practice of cognitive exercises (⩾20 h)		120 (72.3%)	
3. Development of cognitive strategies		125 (75.3%)	
4. Facilitation of transfer to everyday functioning		123 (74.1%)	
4*. Adjunctive psychiatric rehabilitation		48 (28.9%)	
Interventions fulfilling all elements (1.,2.,3.,4*)		36 (21.7%)	

CGI, Clinical Global Impression; CPZeq, chlorpromazine equivalents; CTAM, Clinical Trial Assessment Measure, ITT, Intention-to-treat; PANSS, Positive and Negative Syndrome Scale; IQ, intelligence quotient; s.d., standard deviation; TAU, treatment-as-usual.

CR was compared with treatment as usual (TAU) (61 studies, 36.1%), to active TAU (24 studies, 14.2%), to active non-specific interventions (50 studies, 29.6%), or to active evidence-based interventions implemented for trial purpose (34 studies, 20.1%). Half the studies (80, 53%) were rated as having higher methodological rigor (CTAM score ⩾ 65). Methodological quality improved over time (Spearman *ρ* = 0.279; *p* = 0.001) and was higher in: multicenter studies (Mann–Whitney *U* = 1622.00; *p* < 0.001), larger studies (Spearman *ρ* = 0.450; *p* < 0.001), and those conducted in outpatient settings (Mann–Whitney *U* = 1444.00; *p* = 0.041). Trial quality was also correlated with the number of beneficial CR ingredients included in the intervention (Spearman *ρ* = 0.187; *p* = 0.021), but not to other setting- or treatment-related parameters.

### Acceptability of cognitive remediation

In 31 studies (34 comparisons) no drop-out occurred during treatment phase in any treatment arm. These studies had smaller sample size (Mann–Whitney *U* = 671.00; *p* < 0.001), lower methodological quality (Mann–Whitney *U* = 1106.50; *p* = 0.001), lower treatment duration (Mann–Whitney *U* = 1580.00; *p* = 0.005) and intensity (hours/week, Mann–Whitney *U* = 1362.50; *p* = 0.032), included patients with more severe symptoms (Mann–Whitney *U* = 466.00; *p* = 0.033) and were more often pilot trials (χ^2^ = 7.611, *p* = 0.006).

The overall rate of drop-out was 16.58% for CR programs and 15.21% for control conditions (18.62 and 17.28% respectively when removing trials with no drop-out). In the meta-analysis, no difference emerged between CR interventions and controls regarding attrition: OR 1.10 (95% CI 0.96–1.25), *p* = 0.177. Overall heterogeneity was statistically significant (*Q* = 163.68, df 134, *p* = 0.041), but low (*I*^2^ = 18.13%). The sensitivity analysis conducted using a fixed-effects model yielded very similar results: OR 1.09 (95% CI 0.97–1.22), *p* = 0.132. No evidence of publication bias emerged (no funnel plot asymmetry at visual inspection, Egger test: *p* = 0.591). The results were also comparable when the analysis only included studies where drop-out was reported to be due to low motivation: [OR 1.064 (95% CI 0.821–1.378), *p* = 0.639], although this reduced the sample size (*N* = 36).

### Moderator effects

Drop-out rate was lower in studies recruiting only inpatients compared to studies carried out in outpatient settings or recruiting both outpatients and inpatients [OR 0.88 (95% CI 0.68–1.13) *v.* OR 1.10 (95% CI 0.94–1.29) and 1.67 (95% CI 1.15–2.42), χ^2^ = 7.71, *p* = 0.021]. Attrition rates were similar in pilot/feasibility trials and more rigorous trials [OR 1.05 (95% CI 0.75–1.46) *v.* OR 1.10 (95% CI 0.97–1.24), χ^2^ = 0.07, *p* = 0.793]. These results were robust when restricted only to studies conducted in real-world clinical practice settings [OR 1.07 (95% CI 0.93–1.25), *N* = 93 studies]. See Table 2 for details.

**Table 2.** Effect of moderators

Moderators	Drop-out rate		
	*N* comparisons	Coefficient/OR (CI)	*p*
Study characteristics			
Publication year	135	0.012 (−0.014 a 0.038)	0.358
CTAM score	135	−0.007 (−0.017 a 0.002)	0.137
Methodological quality			
CTAM ⩾65	77	1.05 (0.89 a 1.24)	
CTAM <65	58	1.17 (0.94 a 1.46)	
		χ^2^ = 0.57 (df 1)	0.449
Setting			
Outpatients	86	1.10 (0.94–1.29)	
Inpatients	32	0.88 (0.68–1.13)	
Both	17	1.67 (1.15–2.42)	
		χ^2^ = 7.71 (df 2)	**0.021**
Comparison category			
TAU	44		
Active TAU	19	1.43 (1.10–1.86)	
Nonspecific active control	42	0.71 (0.50–1.03)	
Evidence-based control	30	1.02 (0.80–1.28)	
		1.13 (0.89–1.43)	
		χ^2^ = 9.90 (df 3)	**0.019**
Sample size	135	−0.001 (−0.003 a 0.001)	0.300
Sample composition			
Only participants diagnosed with schizophrenia	54	1.09 (0.92–1.29)	
Other diagnoses included	81	1.11 (0.89–1.39)	
		χ^2^ = 0.03 (df1)	0.873
Payment of included participants			
Present	29	1.19 (0.93–1.51)	
Absent	106	1.07 (0.92–1.25)	
		χ^2^ = 0.49 (df1)	0.483
Participant characteristics			
Age (year)	134	−0.002 (−0.020 a 0.017)	0.847
Gender (%females)	129	0.002 (−0.010 a 0.014)	0.728
Education (years)	93	0.150 (0.039 a 0.262)	**0.008**
Premorbid IQ	53	0.026 (0.002 a 0.050)	**0.033**
Age of onset (years)	88	0.016 (−0.051 a 0.083)	0.644
Duration of illness (years)	91	0.007 (−0.016 a 0.030)	0.553
Baseline therapy dose (CPZeq)	64	0.0002 (−0.001 a 0.001)	0.588
Baseline symptoms severity (PANSS score)	81	0.003 (−0.008 a 0.014)	0.614
Treatment characteristics			
Duration (weeks)	135	−0.002 (−0.009 a 0.005)	0.503
Intensity (season/week)	130	0.032 (−0.061 a 0.126)	0.500
Intensity (hours/week)	126	0.008 (−0.082 a 0.099)	0.856
Active and trained therapist (Element 1)			
Present	107	1.04 (0.91–1.19)	
Absent	28	1.43 (0.98–2.07)	
		χ^2^ = 2.40 (df 1)	0.122
Repeated practice of cognitive exercises (Element 2)			
Present	106	1.11 (0.95–1.29)	
Absent	22	1.09 (0.83–1.43)	
		χ^2^ = 0.02 (df 1)	0.892
Development of cognitive strategies (Element 3)			
Present	102	1.02 (0.88–1.18)	
Absent	33	1.37 (1.02–1.82)	
		χ^2^ = 3.07 (df 1)	0.080
Facilitation of transfer to everyday functioning (Element 4)			
Present	102	1.02 (0.89–1.18)	
Absent	32	1.45 (1.06–1.99)	
		χ^2^ = 4.01 (df1)	**0.045**
Adjunctive psychiatric rehabilitation (Element 4*)			
Present	42	0.91 (0.72–1.15)	
Absent	93	1.19 (1.02–1.40)	
		χ^2^ = 3.50 (df 1)	0.062
Interventions including all core elements (1,2,3,4)			
All core elements	66	0.96 (0.80–1.15)	
Not all core elements included	69	1.25 (1.04–1.50)	
		χ^2^ = 3.99 (df 1)	**0.046**
Interventions including all core elements (1,2,3,4*)			
All core elements	32	0.82 (0.65–1.05)	
Not all core elements included	103	1.20 (1.03–1.40)	
		χ^2^ = 6.83 (df 1)	**0.009**
Format of delivery			
Individual sessions	62	1.25 (1.02–1.54)	
Group sessions	57	0.98 (0.82–1.17)	
Both	16	0.88 (0.59–1.31)	
		χ^2^ = 4.13 (df 2)	0.127
Method of delivery			
Computer-assisted	61	1.05 (0.85–1.31)	
Pencil-and-paper	36	1.12 (0.87–1.44)	
Both	38	1.18 (0.93–1.49)	
		χ^2^ = 0.452 (df 2)	0.798

*C*PZeq, chlorpromazine equivalents; CTAM, Clinical Trial Assessment Measure; PANSS, Positive and Negative Syndrome Scale; IQ, intelligence quotient; TAU, treatment-as-usual.

* = alternative 4^th^ core element.

The type of control had a significant impact (χ^2^ = 9.90, *p* = 0.019) with attrition being higher for CR compared to TAU [OR 1.43 (1.10–1.86)], but there was no consistent difference in comparison to an active control activity or an evidence-based psychosocial intervention. Surprisingly acceptability was higher when the comparison was an active TAU [OR 0.71 (0.50–1.03)]. Methodological quality, publication year, sample size, included diagnoses and payment of participants had no significant effect.

Acceptability of CR interventions was significantly higher in studies with participants showing fewer years of education [Coefficient = 0.150 (0.039–0.262), *p* = 0.008] and lower premorbid IQ [Coefficient = 0.026 (0.002–0.050), *p* = 0.033]. No significant effect was observed for age, gender, age of onset, duration of illness, or baseline symptom severity. Antipsychotic treatment dosage did not emerge as a significant moderator either.

Among the core CR ingredients in our subgroup analyses, the implementation of techniques to transfer cognitive gains to real-world functioning had a significant positive impact on acceptability [Including this element, OR  1.02 (0.89–1.18) not including this element OR 1.45 (1.06–1.99), χ^2^ = 4.01, *p* = 0.045] (see Table 2). The presence of an active and trained therapist, the repetition of cognitive exercises or teaching novel cognitive strategies did not exert a significant effect when considered separately. However, treatments that included all the core ingredients had significantly better acceptability [OR 0.96 (0.80–1.15) *v.* 1.25 (1.04–1.50), χ^2^ = 3.99, *p* = 0.046], which further improved if psychiatric rehabilitation was considered as the optimal transfer technique [OR 0.82 (0.65–1.05) *v.* 1.20 (1.03–1.40), χ^2^ = 6.83, *p* = 0.009]. No significant effect was observed for other treatment-related variables such as duration, intensity, format, and method of treatment delivery.

## Discussion

This is the first systematic and comprehensive assessment of CR acceptability for people living with schizophrenia as measured by the study drop-out rate. The overall drop-out rate (16.58%) was equivalent to that observed in control interventions (15.21%), as confirmed by the quantitative meta-analytic synthesis that found no significant differences between the two groups. Of note, the analysis of drop-out due to low motivation, which can be viewed as the reason more specifically related to the intervention, yielded comparable results. The CR drop-out rate was also comparable to one found across different psychosocial interventions for individuals diagnosed with schizophrenia (Szymczynska et al., 2017; Villeneuve et al., 2010), and lower with respect to drop-out rates reported in pharmacological trials (Bighelli et al., 2020; Cramer & Rosenheck, 1998; Kemmler, Hummer, Widschwendter, & Fleischhacker, 2005; Lieberman & Hsiao, 2006), which often involve shorter observation periods (Bighelli et al., 2020).

Acceptability of CR intervention was higher in studies recruiting inpatients. This result replicates previous literature reporting better feasibility of psychosocial interventions in inpatients setting, as confirmed by a systematic review (Villeneuve et al., 2010). Treatment programs that included all the core ingredients for CR effectiveness (Bowie et al., 2019), and particularly those including techniques to transfer cognitive gains into real-world functioning showed a lower drop-out rate. This is an important finding as it could be linked to a better treatment rationale, improved perceived efficacy by the participants themselves, and may also have positive effects on motivation. Other treatment elements despite having an impact on CR benefits (Vita et al., 2021), did not produce significant effects on acceptability. This result is of practical importance if we are to reduce barriers to successful recovery. Making the treatment rationale clear and linked to functional outcome is part of case formulation and a way of personalizing treatment [see (Wykes & Reeder, 2005) for a suggested method of CR case formulation]. To reduce drop-out (the surrogate for acceptability) and improve the cost-effectiveness of treatment, services choosing a CR intervention as part of their recovery programs should choose one that delivers this element.

The presence of an active and trained therapist did not emerge as a significant factor, despite its emphasis in previous literature (Browne et al., 2021; Fiszdon et al., 2016), although it did have an effect on CR outcomes (Vita et al., 2021). This finding could be explained by therapists enhancing the treatment rationale, encouraging the transfer of CR skill improvement into everyday life and the chances of participants using these skills through the development of a positive therapeutic alliance. This more complex set of relationships requires further analysis.

Participants with lower IQ and fewer years of education displayed better acceptability of CR programs; these characteristics have been already described as important moderators of CR efficacy on both cognition and functioning (Fiszdon, Choi, Bryson, & Bell, 2006; Tan et al., 2019; Twamley, Burton, & Vella, 2011; Vita et al., 2021); a previous study also found a correlation between lower cognitive performance, in particular in attention and working memory domains, and the level of participation to the intervention (Dillon et al., 2016). This finding suggests again that a link might exist between better effectiveness and better acceptability of the intervention. It may be that participants who are more inclined to commit to treatment are those who are more fragile or more compromised and more motivated to obtain an improvement in their clinical and personal condition (Saperstein et al., 2020). This hypothesis, however, remains to be more appropriately and thoroughly investigated.

### Strengths and limitations

One of the main strengths of the present study is its novelty and uniqueness as the first meta-analysis to explore CR intervention acceptability for people living with schizophrenia. The conclusions were drawn from a comprehensive search of the literature and from the largest database reported of CR trials, so the robust results have implications for CR practical implementation.

The most obvious limitation is that we had to adopt drop-out rates as a measure of acceptability because very few studies asked participants what they thought of the treatment. Drop-out from treatment can be affected by many factors, including external life events, change in personal circumstances and symptom changes, although as we were investigating randomized trials these factors would equally occur in the comparison groups. This issue was partially addressed by performing a sub-analysis including only participants who explicitly left the trial due to low motivation. Some heterogeneity also occurred in the study drop-out definitions as they ranged from participants recruited who never received the intervention to those who did not attend the minimum number of treatment sessions.

Engagement in treatment programs might not always coincide with completion rate or sessions attendance (Mahncke et al., 2019): it is important to consider this and other indicators, such as satisfaction with treatment, as crucial complementary outcomes, when interpreting acceptability data derived from indirect proxies such as trial drop-out rates.

Although our findings are based on randomized clinical trials, the majority were conducted in real-world clinical settings and the results the same even after excluding trials in highly controlled or research-oriented sites, so generalization is probable.

The role of pharmacotherapy as a potential moderator of drop-out has been marginally investigated in this study, only expressed as baseline therapy dose (chlorpromazine equivalents). In fact, concomitant antipsychotic treatment could be involved in either jeopardizing or enhancing the effects of CR (Biagianti, Castellaro, & Brambilla, 2021). Further exploring the role of pharmacotherapy in influencing the acceptability of CR, which did not emerge as significant in the current analyses, as well as the eventual role of medication adherence (Nuechterlein et al., 2020), could represent a relevant target for future studies. In this light, it should be pointed out that only a few details on concomitant pharmacotherapy were reported in studies included in the present review, and this issue seems rather frequent in CR trials; besides, it could be better explored using a different methodology for quantitative synthesis of results, such as individual patient data meta-analyses.

Analyzing the influence of pharmacological treatment on adherence to non-pharmacological interventions represents an interesting topic, also considering the growing literature interest for providing cognitive enhancement pharmacological strategies (Fleischhacker et al., 2021; Harvey & Sand, 2017; Michalopoulou et al., 2015; Otto et al., 2016).

### Future studies

We urge two things of our scientist colleagues. To agree on the measure of drop-out as this will make it easier to compare different treatments and then make recommendations to national healthcare services. But just as vital is asking about acceptability directly, as the patient voice should be heard before making key decisions about treatment provision. A further recommendation is about the measurement of outcome in CR trials. We adopt a primary outcome measure and other assessments are then secondary. If a more nuanced approach was implemented then we could use the value placed by different groups (service providers, clinicians, and service users) and use a combined measure of the worth of treatment. These methods have rarely been used and are called Multi-Criterion Decision Modeling (MCDM) (Cinelli, Kadziński, Gonzalez, & Słowiński, 2020; Greco, Figueira, & Ehrgott, 2016), but would balance what are sometimes competing views of different parts of our health care system. Drop-out rates or satisfaction with treatment might be one element of an MCDM.

## Conclusions

CR is an evidence-based intervention for people living with schizophrenia that is not only effective in producing cognitive and functional gains, but is also characterized by a good acceptability profile, similar to that of other evidence-based psychosocial interventions, and superior to that of pharmacological treatments. One core CR ingredient had an impact on acceptability, facilitation of transfer to everyday functioning. Other key elements also contribute to benefits, but this one is of great value in clinical practice as it values the subjective recovery preferences of people living with schizophrenia.

## Supplementary material

For supplementary material accompanying this paper visit https://doi.org/10.1017/S0033291722000319. click here to view supplementary material
