# Supplementary material for: Acceptability of cognitive remediation for schizophrenia: a systematic review and meta-analysis of randomized controlled trials
Source: Psychol Med. 2022 Mar 8;53(8):3661–71. doi: 10.1017/S0033291722000319 (PMC10277755; doi:10.1017/S0033291722000319)
Supplement: Supplementary file 1 [file S0033291722000319sup.zip › S0033291722000319sup001.docx]

**Appendix 1: Moderator effects**

Methodology of included studies: publication year, overall methodological quality, presence of blinding, use of intention-to-treat approaches, comparison category (Treatment As Usual-TAU, e.g. drug treatment/case management, waiting lists, TAU with no description provided; active TAU -including multidisciplinary rehabilitative programs; active nonspecific interventions controlling for nonspecific aspects and matched with CR for duration and schedule -e.g. social stimulation, leisure activities, computer activities; active evidence-based interventions), inclusion of diagnoses besides schizophrenia, study setting (real-world clinical practice or research facility/academic setting).

Characteristics of included interventions: presence of the 4 core elements of CR (presence of an active and trained therapist, repeated practice of cognitive exercises, structured development of cognitive strategies, and use of techniques to improve the transfer of cognitive gains to the real world), format of delivery, computer use, treatment duration (in weeks) and intensity (in sessions per week and hours per week).

Study participants: age, sex (expressed as percentages of female participants), years of education, premorbid IQ, age at onset, duration of illness, baseline treatment dosage (chlorpromazine equivalents) and baseline symptom severity (PANSS score).
