# Supplementary material for: Acceptability of cognitive remediation for schizophrenia: a systematic review and meta-analysis of randomized controlled trials
Source: Psychol Med. 2022 Mar 8;53(8):3661–71. doi: 10.1017/S0033291722000319 (PMC10277755; doi:10.1017/S0033291722000319)
Supplement: Supplementary file 1 [file S0033291722000319sup.zip › S0033291722000319sup002.docx]

**Appendix 2: Included studies**

1. Aghotor J, Pfueller U, Moritz S, Weisbrod M, Roesch-Ely D. Metacognitive training for patients with schizophrenia (MCT): feasibility and preliminary evidence for its efficacy. J Behav Ther Exp Psychiatry 2010; 41: 207–11.
2. Ahmed AO, Hunter KM, Goodrum NM, et al. A randomized study of cognitive remediation for forensic and mental health patients with schizophrenia. J Psychiatr Res 2015; 68: 8–18.
3. Ahuir M, Cabezas Á, Miñano MJ, et al. Improvement in cognitive biases after group psychoeducation and metacognitive training in recent-onset psychosis: A randomized crossover clinical trial. Psychiatry Res 2018; 270: 720–3.
4. Aloi M, de Filippis R, Grosso Lavalle F, et al. Effectiveness of integrated psychological therapy on clinical, neuropsychological, emotional and functional outcome in schizophrenia: a RCT study. J Ment Health Abingdon Engl 2018; : 1–8.
5. Au DWH, Tsang HWH, So WWY, et al. Effects of integrated supported employment plus cognitive remediation training for people with schizophrenia and schizoaffective disorders. Schizophr Res 2015; 166: 297–303.
6. Bell M, Bryson G, Greig T, Corcoran C, Wexler BE. Neurocognitive enhancement therapy with work therapy: effects on neuropsychological test performance. Arch Gen Psychiatry 2001; 58: 763–8.
7. Bellucci DM, Glaberman K, Haslam N. Computer-assisted cognitive rehabilitation reduces negative symptoms in the severely mentally ill. Schizophr Res 2003; 59: 225–32.
8. Bosia M, Bechi M, Marino E, et al. Influence of catechol-O-methyltransferase Val158Met polymorphism on neuropsychological and functional outcomes of classical rehabilitation and cognitive remediation in schizophrenia. Neurosci Lett 2007; 417(3):271-4.
9. Bowie CR, McGurk SR, Mausbach B, Patterson TL, Harvey PD. Combined cognitive remediation and functional skills training for schizophrenia: effects on cognition, functional competence, and real-world behavior. Am J Psychiatry 2012; 169: 710–8.
10. Briki M, Monnin J, Haffen E, et al. Metacognitive training for schizophrenia: a multicentre randomised controlled trial. Schizophr Res 2014; 157: 99–106.
11. Bryce SD, Rossell SL, Lee SJ, et al. Neurocognitive and Self-efficacy Benefits of Cognitive Remediation in Schizophrenia: A Randomized Controlled Trial. J Int Neuropsychol Soc JINS 2018; 24: 549–62.
12. Bucci P, Piegari G, Mucci A, et al. Neurocognitive individualized training versus social skills individualized training: a randomized trial in patients with schizophrenia. Schizophr Res 2013; 150(1):69-75.
13. Burda PC, Starkey TW, Dominguez F, Vera V. Computer-assisted cognitive rehabilitation of chronic psychiatric inpatients. Comput Hum Behav 1994; 10: 359–68.
14. Byrne LK, Peng D, McCabe M, et al. Does practice make perfect? Results from a Chinese feasibility study of cognitive remediation in schizophrenia. Neuropsychol Rehabil 2013; 23: 580–96.
15. Cassetta BD, Tomfohr-Madsen LM, Goghari VM. A randomized controlled trial of working memory and processing speed training in schizophrenia. Psychol Med 2019; 49: 2009–19.
16. Cavallaro R, Anselmetti S, Poletti S, et al. Computer-aided neurocognitive remediation as an enhancing strategy for schizophrenia rehabilitation. Psychiatry Res 2009; 169: 191–6.
17. Cavallo M, Trivelli F, Adenzato M, et al. Do neuropsychological and social cognition abilities in schizophrenia change after intensive cognitive training? A pilot study. Clin Neuropsychiatr 2013; 10(5): 202-11.
18. Choi K-H, Kwon J-H. Social cognition enhancement training for schizophrenia: a preliminary randomized controlled trial. Community Ment Health J 2006; 42: 177–87.
19. Choi K-H, Kang J, Kim S-M, et al. Cognitive Remediation in Middle-Aged or Older Inpatients with Chronic Schizophrenia: A Randomized Controlled Trial in Korea. Front Psychol 2017; 8: 2364.
20. Choi J, Taylor B, Fiszdon JM, et al. The synergistic benefits of physical and cognitive exercise in schizophrenia: Promoting motivation to enhance community effectiveness. Schizophr Res Cogn. 2019; 19:100147.
21. d’Amato T, Bation R, Cochet A, et al. A randomized, controlled trial of computer-assisted cognitive remediation for schizophrenia. Schizophr Res 2011; 125: 284–90.
22. Dark F, Scotti JC, Baker A, et al. Randomized controlled trial of social cognition and interaction training compared to befriending group. Br J Clin Psychol 2020; 59(3):384-402.
23. De Pinho LMG, da Cruz Sequeira CA, Correia Sampaio FM, et al. Assessing the efficacy and feasibility of providing metacognitive training for patients with schizophrenia by mental health nurses: A randomized controlled trial. J Adv Nurs 2021; 77(2):999-1012.
24. D’Souza DC, Radhakrishnan R, Perry E, et al. Feasibility, Safety, and Efficacy of the Combination of 𝒟-Serine and Computerized Cognitive Retraining in Schizophrenia: An International Collaborative Pilot Study. Neuropsychopharmacology 2013; 38: 492–503.
25. Dickinson D, Tenhula W, Morris S, et al. A randomized, controlled trial of computer-assisted cognitive remediation for schizophrenia. Am J Psychiatry 2010; 167: 170–80.
26. Donohoe G, Dillon R, Hargreaves A, et al. Effectiveness of a low support, remotely accessible, cognitive remediation training programme for chronic psychosis: cognitive, functional and cortical outcomes from a single blind randomised controlled trial. Psychol Med 2018; 48: 751–64.
27. Drake RJ, Day CJ, Picucci R, et al. A naturalistic, randomized, controlled trial combining cognitive remediation with cognitive-behavioural therapy after first-episode non-affective psychosis. Psychol Med 2014; 44: 1889–99.
28. Eack SM, Greenwald DP, Hogarty SS, et al. Cognitive enhancement therapy for early-course schizophrenia: effects of a two-year randomized controlled trial. Psychiatr Serv Wash DC 2009; 60: 1468–76.
29. Eack SM, Hogarty SS, Greenwald DP, et al. Cognitive Enhancement Therapy in substance misusing schizophrenia: results of an 18-month feasibility trial. Schizophr Res 2015;161(2-3):478-83.
30. Estrada F, Crosas JM, Ahuir M, et al. Free Thyroxine Concentrations Moderate the Response to a Cognitive Remediation Therapy in People With Early Psychosis: A Pilot Randomized Clinical Trial. Front Psychiatry 2020; 11:636.
31. Fan F, Zou Y, Tan Y, Hong LE, Tan S. Computerized cognitive remediation therapy effects on resting state brain activity and cognition in schizophrenia. Sci Rep 2017; 7. DOI:10.1038/s41598-017-04829-9.
32. Farreny A, Aguado J, Ochoa S, et al. REPYFLEC cognitive remediation group training in schizophrenia: Looking for an integrative approach. Schizophr Res 2012; 142: 137–44.
33. Favrod J, Rexhaj S, Bardy S, et al. Sustained antipsychotic effect of metacognitive training in psychosis: a randomized-controlled study. Eur Psychiatry J Assoc Eur Psychiatr 2014; 29: 275–81.
34. Fernandez-Gonzalo S, Turon M, Jodar M, et al. A new computerized cognitive and social cognition training specifically designed for patients with schizophrenia/schizoaffective disorder in early stages of illness: A pilot study. Psychiatry Res 2015; 228: 501–9.
35. Fisher M, Loewy R, Carter C, et al. Neuroplasticity-Based Auditory Training Via Laptop Computer Improves Cognition in Young Individuals With Recent Onset Schizophrenia. Schizophr Bull 2015; 41: 250–8.
36. Fisher M, Mellon SH, Wolkowitz O, Vinogradov S. Neuroscience-informed Auditory Training in Schizophrenia: A Final Report of the Effects on Cognition and Serum Brain-Derived Neurotrophic Factor. Schizophr Res Cogn 2016; 3: 1–7.
37. Fiszdon JM, Choi KH, Bell MD, Choi J, Silverstein SM. Cognitive remediation for individuals with psychosis: efficacy and mechanisms of treatment effects. Psychol Med 2016; 46: 3275–89.
38. Galderisi S, Piegari G, Mucci A, et al. Social skills and neurocognitive individualized training in schizophrenia: comparison with structured leisure activities. Eur Arch Psychiatry Clin Neurosci 2010; 260: 305–15.
39. García S, Fuentes I, Ruíz JC, Gallach E, Roder V. Application of the IPT in a Spanish sample: Evaluation of the ‘social perception subprogramme’. Int J Psychol Psychol Ther 2003; 3: 299–310.
40. García-Fernández L, Cabot-Ivorra N, Rodríguez-García V, et al. Computerized cognitive remediation therapy, REHACOM, in first episode of schizophrenia: A randomized controlled trial. Psychiatry Res 2019; 281: 112563.
41. Garrido G, Barrios M, Penadés R, et al. Computer-assisted cognitive remediation therapy: cognition, self-esteem and quality of life in schizophrenia. Schizophr Res 2013; 150: 563–9.
42. Gawęda Ł, Krężołek M, Olbryś J, Turska A, Kokoszka A. Decreasing self-reported cognitive biases and increasing clinical insight through meta-cognitive training in patients with chronic schizophrenia. J Behav Ther Exp Psychiatry 2015; 48: 98–104.
43. Gharaeipour M, Scott BJ. Effects of cognitive remediation on neurocognitive functions and psychiatric symptoms in schizophrenia inpatients. Schizophr Res 2012; 142: 165–70.
44. Gohar SM, Hamdi E, El Ray LA, Horan WP, Green MF. Adapting and evaluating a social cognitive remediation program for schizophrenia in Arabic. Schizophr Res 2013; 148: 12–7.
45. Gomar JJ, Valls E, Radua J, et al. A Multisite, Randomized Controlled Clinical Trial of Computerized Cognitive Remediation Therapy for Schizophrenia. Schizophr Bull 2015; 41: 1387–96.
46. Gordon A, Davis PJ, Patterson S, et al. A randomized waitlist control community study of Social Cognition and Interaction Training for people with schizophrenia. Br J Clin Psychol 2018; 57: 116–30.
47. Greig TC, Zito W, Wexler BE, Fiszdon J, Bell MD. Improved cognitive function in schizophrenia after one year of cognitive training and vocational services. Schizophr Res 2007; 96: 156–61.
48. Guimond S, Ling G, Drodge J, et al. Functional connectivity associated with improvement in emotion management after cognitive enhancement therapy in early-course schizophrenia. Psychol Med 2020; 1-10.
49. Habel U, Koch K, Kellermann T, et al. Training of affect recognition in schizophrenia: Neurobiological correlates. Soc Neurosci 2010; 5: 92–104.
50. Hadas-Lidor N, Katz N, Tyano S, Weizman A. Effectiveness of dynamic cognitive intervention in rehabilitation of clients with schizophrenia. Clin Rehabil 2001; 15: 349–59.
51. Hegde S, Rao SL, Raguram A, Gangadhar BN. Addition of home-based cognitive retraining to treatment as usual in first episode schizophrenia patients: a randomized controlled study. Indian J Psychiatry 2012; 54: 15–22.
52. Hermanutz M, Gestrich J. Computer-assisted attention training in schizophrenics. A comparative study. Eur Arch Psychiatry Clin Neurosci 1991; 240: 282–7.
53. Hodge MAR, Siciliano D, Withey P, et al. A Randomized Controlled Trial of Cognitive Remediation in Schizophrenia. Schizophr Bull 2010; 36: 419–27.
54. Hogarty GE, Flesher S, Ulrich R, et al. Cognitive enhancement therapy for schizophrenia: effects of a 2-year randomized trial on cognition and behavior. Arch Gen Psychiatry 2004; 61: 866–76.
55. Hooker CI, Bruce L, Fisher M, Verosky SC, Miyakawa A, Vinogradov S. Neural activity during emotion recognition after combined cognitive plus social cognitive training in schizophrenia. Schizophr Res 2012; 139: 53–9.
56. Horan WP, Kern RS, Shokat-Fadai K, Sergi MJ, Wynn JK, Green MF. Social cognitive skills training in schizophrenia: an initial efficacy study of stabilized outpatients. Schizophr Res 2009; 107: 47–54.
57. Horan WP, Kern RS, Tripp C, et al. Efficacy and specificity of Social Cognitive Skills Training for outpatients with psychotic disorders. J Psychiatr Res 2011; 45: 1113–22.
58. Horan WP, Dolinsky M, Lee J, et al. Social Cognitive Skills Training for Psychosis With Community-Based Training Exercises: A Randomized Controlled Trial. Schizophr Bull 2018; 44: 1254–66.
59. Iwata K, Matsuda Y, Sato S, et al. Efficacy of cognitive rehabilitation using computer software with individuals living with schizophrenia: A randomized controlled trial in Japan. Psychiatr Rehabil J 2017; 40: 4–11.
60. Jahshan C, Vinogradov S, Wynn JK, Hellemann G, Green MF. A randomized controlled trial comparing a ‘bottom-up’ and ‘top-down’ approach to cognitive training in schizophrenia. J Psychiatr Res 2019; 109: 118–25.
61. Kanie A, Kikuchi A, Haga D, et al. The Feasibility and Efficacy of Social Cognition and Interaction Training for Outpatients With Schizophrenia in Japan: A Multicenter Randomized Clinical Trial. Front Psychiatry 2019; 10: 589.
62. Kantrowitz JT, Sharif Z, Medalia A, et al. A Multicenter, Rater-Blinded, Randomized Controlled Study of Auditory Processing-Focused Cognitive Remediation Combined With Open-Label Lurasidone in Patients With Schizophrenia and Schizoaffective Disorder. J Clin Psychiatry 2016; 77: 799–806.
63. Kariofillis D, Sartory G, Kärgel C, Müller BW. The effect of cognitive training on evoked potentials in schizophrenia. Schizophr Res Cogn. 2014; 1(4):180-186.
64. Katsumi A, Hoshino H, Fujimoto S, et al. Effects of cognitive remediation on cognitive and social functions in individuals with schizophrenia. Neuropsychol Rehabil 2019; 29: 1475–87.
65. Keefe RSE, Vinogradov S, Medalia A, et al. Feasibility and pilot efficacy results from the multisite Cognitive Remediation in the Schizophrenia Trials Network (CRSTN) randomized controlled trial. J Clin Psychiatry 2012; 73: 1016–22.
66. Kidd SA, Kaur J, Virdee G, George TP, McKenzie K, Herman Y. Cognitive remediation for individuals with psychosis in a supported education setting: a randomized controlled trial. Schizophr Res 2014; 157: 90–8.
67. Kidd SA, Herman Y, Virdee G, Bowie CR, et al. A comparison of compensatory and restorative cognitive interventions in early psychosis. Schizophr Res Cogn. 2019; 19:100157.
68. Klingberg S, Wölwer W, Engel C, et al. Negative symptoms of schizophrenia as primary target of cognitive behavioral therapy: results of the randomized clinical TONES study. Schizophr Bull 2011; 37 Suppl 2: S98-110.
69. Krzystanek M, Borkowski M, Skałacka K, Krysta K. A telemedicine platform to improve clinical parameters in paranoid schizophrenia patients: Results of a one-year randomized study. Schizophr Res; 204:389-396.
70. Kukla M, Bell MD, Lysaker PH. A randomized controlled trial examining a cognitive behavioral therapy intervention enhanced with cognitive remediation to improve work and neurocognition outcomes among persons with schizophrenia spectrum disorders. Schizophr Res 2018; 197: 400–6.
71. Kumar D, Haq MZU, Dubey I, et al. Effect of meta-cognitive training in the reduction of positive symptoms in schizophrenia. Eur J Psychother Couns 2010; 12: 149–58.
72. Kuokkanen R, Lappalainen R, Repo-Tiihonen E, Tiihonen J. Metacognitive group training for forensic and dangerous non-forensic patients with schizophrenia: a randomised controlled feasibility trial. Crim Behav Ment Health. 2014; 24(5):345-57.
73. Kurtz MM, Seltzer JC, Shagan DS, Thime WR, Wexler BE. Computer-Assisted Cognitive Remediation in Schizophrenia: What is the Active Ingredient? Schizophr Res 2007; 89: 251–60.
74. Kurtz MM, Mueser KT, Thime WR, Corbera S, Wexler BE. Social Skills Training and Computer-Assisted Cognitive Remediation in Schizophrenia. Schizophr Res 2015; 162: 35–41.
75. Lado-Codesido M, Pérez CM, Mateos R, Olivares JM, Caballero AG. Improving emotion recognition in schizophrenia with ‘VOICES’: An on-line prosodic self-training. PLOS ONE 2019; 14: e0210816.
76. Lam KCK, Ho CPS, Wa JC, et al. Metacognitive training (MCT) for schizophrenia improves cognitive insight: a randomized controlled trial in a Chinese sample with schizophrenia spectrum disorders. Behav Res Ther 2015; 64:38-42.
77. Lee WK. Effectiveness of computerized cognitive rehabilitation training on symptomatological, neuropsychological and work function in patients with schizophrenia. Asia-Pac Psychiatry 2013; 5: 90–100.
78. Lindenmayer J-P, McGurk SR, Mueser KT, et al. A randomized controlled trial of cognitive remediation among inpatients with persistent mental illness. Psychiatr Serv Wash DC 2008; 59: 241–7.
79. Lu H, Li Y, Li F, et al. Randomized controlled trial on adjunctive cognitive remediation therapy for chronically hospitalized patients with schizophrenia. Shanghai Arch Psychiatry 2012; 24: 149–54.
80. Mahncke HW, Kim S-J, Rose A, et al. Evaluation of a plasticity-based cognitive training program in schizophrenia: Results from the eCaesar trial. Schizophr Res 2019; 208: 182–9.
81. Mak M, Samochowiec J, Tybura P, et al. The efficacy of cognitive rehabilitation with RehaCom programme in schizophrenia patients. The role of selected genetic polymorphisms in successful cognitive rehabilitation. Ann Agric Environ Med 2013; 20. http://yadda.icm.edu.pl/yadda/element/bwmeta1.element.agro-f8ce606e-213f-45cd-9ff1-80554003aef0 (accessed May 13, 2020).
82. Man DWK, Law KM, Chung RCK. Cognitive training for Hong Kong Chinese with schizophrenia in vocational rehabilitation. Hong Kong Med J Xianggang Yi Xue Za Zhi 2012; 18 Suppl 6: 18–22.
83. Maroño Souto Y, Vázquez Campo M, Díaz Llenderrozas F, Rodríguez Álvarez M, Mateos R, García Caballero A. Randomized Clinical Trial with e-MotionalTraining® 1.0 for Social Cognition Rehabilitation in Schizophrenia. Front Psychiatry 2018; 9: 40.
84. Matsuda Y, Morimoto T, Furukawa S, et al. Feasibility and effectiveness of a cognitive remediation programme with original computerised cognitive training and group intervention for schizophrenia: a multicentre randomised trial. Neuropsychol Rehabil 2018; 28: 387–97.
85. Matsui M, Arai H, Yonezawa M, Sumiyoshi T, Suzuki M, Kurachi M. The effects of cognitive rehabilitation on social knowledge in patients with schizophrenia. Appl Neuropsychol 2009; 16: 158–64.
86. McGurk SR, Mueser KT, Pascaris A. Cognitive training and supported employment for persons with severe mental illness: one-year results from a randomized controlled trial. Schizophr Bull 2005; 31: 898–909.
87. McGurk SR, Mueser KT, Xie H, et al. Cognitive remediation for vocational rehabilitation nonresponders. Schizophr Res 2016; 175: 48–56.
88. Medalia A, Aluma M, Tryon W, Merriam AE. Effectiveness of attention training in schizophrenia. Schizophr Bull 1998; 24: 147–52.
89. Medalia A, Revheim N, Casey M. Remediation of memory disorders in schizophrenia. Psychol Med 2000; 30: 1451–9.
90. Meichenbaum D, Cameron R. Training schizophrenics to talk to themselves: A means of developing attentional controls. Behav Ther 1973; 4: 515–34.
91. Mendella PD, Burton CZ, Tasca GA, Roy P, St Louis L, Twamley EW. Compensatory cognitive training for people with first-episode schizophrenia: results from a pilot randomized controlled trial. Schizophr Res 2015; 162: 108–11.
92. Mohammadi F, Momtaz YA, Motallebi SA, et al. The Effect of Cognitive Remediation Therapy on Social Skills in Institutionalized Elderly Patients with Schizophrenia. Rev Recent Clin Trials 2017;12(3):182-186.
93. Morimoto T, Matsuda Y, Matsuoka K, et al. Computer-assisted cognitive remediation therapy increases hippocampal volume in patients with schizophrenia: a randomized controlled trial. BMC Psychiatry 2018; 18. DOI:10.1186/s12888-018-1667-1.
94. Moritz S, Kerstan A, Veckenstedt R, et al. Further evidence for the efficacy of a metacognitive group training in schizophrenia. Behav Res Ther 2011; 49: 151–7.
95. Moritz S, Thoering T, Kühn S, Willenborg B, Westermann S, Nagel M. Metacognition-augmented cognitive remediation training reduces jumping to conclusions and overconfidence but not neurocognitive deficits in psychosis. Front Psychol 2015; 6. DOI:10.3389/fpsyg.2015.01048.
96. Müller DR, Schmidt SJ, Roder V. One-year randomized controlled trial and follow-up of integrated neurocognitive therapy for schizophrenia outpatients. Schizophr Bull 2015; 41: 604–16.
97. Müller DR, Khalesi Z, Benzing V, et al. Does Integrated Neurocognitive Therapy (INT) reduce severe negative symptoms in schizophrenia outpatients? Schizophr Res 2017; 188: 92–7.
98. Müller DR, Khalesi Z, Roder V. Can cognitive remediation in groups prevent relapse? Results of a 1-year follow-up randomized controlled trial. J Nerv Ment Dis 2020; 208(5): 362-70.
99. Nahum M, Lee H, Fisher M, et al. Online Social Cognition Training in Schizophrenia: A Double-Blind, Randomized, Controlled Multi-Site Clinical Trial. Schizophr Bull 2021;47(1):108-117.
100. Obeid S, Hallit S, Sacre H, Kazour GS. Effectiveness of integrated psychological therapy on cognitive function among Lebanese patients with schizophrenia: a pilot study. Int J Psychiatry Clin Pract 2020; 24(1):43-52.
101. Ochoa S, López-Carrilero R, Barrigón ML, et al. Randomized control trial to assess the efficacy of metacognitive training compared with a psycho-educational group in people with a recent-onset psychosis. Psychol Med 2017; 47: 1573–84.
102. Ojeda N, Peña J, Sánchez P, et al. Efficiency of cognitive rehabilitation with REHACOP in chronic treatment resistant Hispanic patients. NeuroRehabilitation 2012; 30: 65–74.
103. Omiya H, Yamashita K, Miyata T, et al. Pilot Study of the Effects of Cognitive Remediation Therapy Using the Frontal/Executive Program for Treating Chronic Schizophrenia. Open Psychol J 2016; 09. DOI:10.2174/1874350101609010121.
104. O’Reilly K, Donohoe G, O’Sullivan D, et al. A randomized controlled trial of cognitive remediation for a national cohort of forensic patients with schizophrenia or schizoaffective disorder. BMC Psychiatry 2019; 19: 27.
105. Østergaard Christensen T, Vesterager L, Krarup G, et al. Cognitive remediation combined with an early intervention service in first episode psychosis. Acta Psychiatr Scand 2014; 130: 300–10.
106. Park S, Lee HK, Kim H. Effects of a Korean version of the metacognitive training program for outpatients with schizophrenia on theory of mind, positive symptoms, and interpersonal relationships. Behav Cogn Psychother 2020; 48(1):14-24.
107. Peña J, Ibarretxe-Bilbao N, Sánchez P, et al. Combining social cognitive treatment, cognitive remediation, and functional skills training in schizophrenia: a randomized controlled trial. Npj Schizophr 2016; 2: 1–7.
108. Penadés R, Catalán R, Salamero M, et al. Cognitive remediation therapy for outpatients with chronic schizophrenia: a controlled and randomized study. Schizophr Res 2006; 87: 323–31.
109. Penadés R, López-Vílchez I, Catalán R, et al. BDNF as a marker of response to cognitive remediation in patients with schizophrenia: A randomized and controlled trial. Schizophr Res 2018; 197: 458–64.
110. Pontes LMM, Martins CB, Napolitano IC, et al. Cognitive Training for Schizophrenia in Developing Countries: A Pilot Trial in Brazil. Schizophr Res Treat 2013; 2013. DOI:10.1155/2013/321725.
111. Popova P, Popov TG, Wienbruch C, Carolus AM, Miller GA, Rockstroh BS. Changing facial affect recognition in schizophrenia: Effects of training on brain dynamics. NeuroImage Clin 2014; 6: 156–65.
112. Puig O, Penadés R, Baeza I, et al. Cognitive remediation therapy in adolescents with early-onset schizophrenia: a randomized controlled trial. J Am Acad Child Adolesc Psychiatry 2014; 53: 859–68.
113. Rakitzi S, Georgila P, Efthimiou K, Mueller DR. Efficacy and feasibility of the Integrated Psychological Therapy for outpatients with schizophrenia in Greece: Final results of a RCT. Psychiatry Res 2016; 242: 137–43.
114. Ramsay IS, Nienow TM, Marggraf MP, MacDonald AW. Neuroplastic changes in patients with schizophrenia undergoing cognitive remediation: triple-blind trial. Br J Psychiatry J Ment Sci 2017; 210: 216–22.
115. Rass O, Forsyth JK, Bolbecker AR, et al. Computer-assisted cognitive remediation for schizophrenia: a randomized single-blind pilot study. Schizophr Res 2012; 139: 92–8.
116. Reeder C, Huddy V, Cella M, et al. A new generation computerised metacognitive cognitive remediation programme for schizophrenia (CIRCuiTS): a randomised controlled trial. Psychol Med 2017; 47: 2720–30.
117. Roberts DL, Combs DR, Willoughby M, et al. A randomized, controlled trial of Social Cognition and Interaction Training (SCIT) for outpatients with schizophrenia spectrum disorders. Br J Clin Psychol 2014; 53: 281–98.
118. Rocha NB, Campos C, Figueiredo JM, et al. Social cognition and interaction training for recent-onset schizophrenia: A preliminary randomized trial. Early Interv Psychiatry 2021; 15(1):206-212.
119. Roncone R, Mazza M, Frangou I, et al. Rehabilitation of theory of mind deficit in schizophrenia: a pilot study of metacognitive strategies in group treatment. 2004; published online Sept. DOI:info:doi/10.1080/09602010343000291.
120. Royer A, Grosselin A, Bellot C, et al. Is there any impact of cognitive remediation on an ecological test in schizophrenia? Cognit Neuropsychiatry 2012; 17: 19–35.
121. Sachs G, Winklbaur B, Jagsch R, et al. Training of affect recognition (TAR) in schizophrenia--impact on functional outcome. Schizophr Res 2012; 138: 262–7.
122. Sánchez P, Peña J, Bengoetxea E, et al. Improvements in negative symptoms and functional outcome after a new generation cognitive remediation program: a randomized controlled trial. Schizophr Bull 2014; 40: 707–15.
123. Sartory G, Zorn C, Groetzinger G, Windgassen K. Computerized cognitive remediation improves verbal learning and processing speed in schizophrenia. Schizophr Res 2005; 75: 219–23.
124. Sevos J, Grosselin A, Gauthier M, Carmona F, Gay A, Massoubre C. Cinemotion, a Program of Cognitive Remediation to Improve the Recognition and Expression of Facial Emotions in Schizophrenia: A Pilot Study. Front Psychiatry 2018; 9. DOI:10.3389/fpsyt.2018.00312.
125. Shan X, Liao R, Ou Y, et al. Metacognitive Training Modulates Default-Mode Network Homogeneity During 8-Week Olanzapine Treatment in Patients With Schizophrenia. Front Psychiatry 2020; 11:234.
126. Silverstein SM, Spaulding WD, Menditto AA, et al. Attention Shaping: a Reward-Based Learning Method to Enhance Skills Training Outcomes in Schizophrenia. Schizophr Bull 2009; 35: 222–32.
127. So SH-W, Chan AP, Chong CS-Y, et al. Metacognitive training for delusions (MCTd): effectiveness on data-gathering and belief flexibility in a Chinese sample. Front Psychol 2015; 6: 730.
128. So SH-W, Chan GH-K, Wong CK-W, et al. A randomised controlled trial of metacognitive training for psychosis, depression, and belief flexibility. J Affect Disord 2021; 279:388-397.
129. Spaulding WD, Reed D, Sullivan M, Richardson C, Weiler M. Effects of cognitive treatment in psychiatric rehabilitation. Schizophr Bull 1999; 25: 657–76.
130. Tan B-L, King R. The effects of cognitive remediation on functional outcomes among people with schizophrenia: a randomised controlled study. Aust N Z J Psychiatry 2013; 47: 1068–80.
131. Tan S, Zou Y, Wykes T, et al. Group cognitive remediation therapy for chronic schizophrenia: A randomized controlled trial. Neurosci Lett 2016; 626: 106–11.
132. Tan S, Zhu X, Fan H, et al. Who will benefit from computerized cognitive remediation therapy? Evidence from a multisite randomized controlled study in schizophrenia. Psychol Med 2019; : 1–11.
133. Tao J, Zeng Q, Liang J, Zhou A, Yin X, Xu A. Effects of cognitive rehabilitation training on schizophrenia: 2 years of follow-up. Int J Clin Exp Med 2015; 8: 16089–94.
134. Tas C, Danaci AE, Cubukcuoglu Z, Brüne M. Impact of family involvement on social cognition training in clinically stable outpatients with schizophrenia -- a randomized pilot study. Psychiatry Res 2012; 195: 32–8.
135. Thomas ML, Bismark AW, Joshi YB, et al. Targeted cognitive training improves auditory and verbal outcomes among treatment refractory schizophrenia patients mandated to residential care. Schizophr Res 2018; 202: 378–84.
136. Twamley EW, Vella L, Burton CZ, Heaton RK, Jeste DV. Compensatory cognitive training for psychosis: effects in a randomized controlled trial. J Clin Psychiatry 2012; 73: 1212–9.
137. Ueland T, Rund BR. A controlled randomized treatment study: the effects of a cognitive remediation program on adolescents with early onset psychosis. Acta Psychiatr Scand 2004; 109: 70–4.
138. van Oosterhout B, Krabbendam L, de Boer K, et al. Metacognitive group training for schizophrenia spectrum patients with delusions: a randomized controlled trial. Psychol Med 2014; 44: 3025–35.
139. Vaskinn A, Løvgren A, Egeland MK, et al. A randomized controlled trial of training of affect recognition (TAR) in schizophrenia shows lasting effects for theory of mind. Eur Arch Psychiatry Clin Neurosci 2019; 269: 611–20.
140. Vauth R, Corrigan PW, Clauss M, et al. Cognitive strategies versus self-management skills as adjunct to vocational rehabilitation. Schizophr Bull 2005; 31: 55–66.
141. Ventura J, Subotnik KL, Gretchen-Doorly D, et al. Cognitive remediation can improve negative symptoms and social functioning in first-episode schizophrenia: A randomized controlled trial. Schizophr Res 2019; 203: 24–31.
142. Vidarsdottir OG, Roberts DL, Twamley EW, Gudmundsdottir B, Sigurdsson E, Magnusdottir BB. Integrative cognitive remediation for early psychosis: Results from a randomized controlled trial. Psychiatry Res 2019; 273: 690–8.
143. Vita A, De Peri L, Barlati S, et al. Effectiveness of different modalities of cognitive remediation on symptomatological, neuropsychological, and functional outcome domains in schizophrenia: a prospective study in a real-world setting. Schizophr Res 2011; 133: 223–31.
144. Vita A, De Peri L, Barlati S, et al. Psychopathologic, neuropsychological and functional outcome measures during cognitive rehabilitation in schizophrenia: a prospective controlled study in a real-world setting. Eur Psychiatry J Assoc Eur Psychiatr 2011; 26: 276–83.
145. Wölwer W, Frommann N, Halfmann S, Piaszek A, Streit M, Gaebel W. Remediation of impairments in facial affect recognition in schizophrenia: efficacy and specificity of a new training program. Schizophr Res 2005; 80: 295–303.
146. Wykes T, Reeder C, Corner J, Williams C, Everitt B. The effects of neurocognitive remediation on executive processing in patients with schizophrenia. Schizophr Bull 1999; 25: 291–307.
147. Wykes T, Newton E, Landau S, Rice C, Thompson N, Frangou S. Cognitive remediation therapy (CRT) for young early onset patients with schizophrenia: an exploratory randomized controlled trial. Schizophr Res 2007; 94: 221–30.
148. Wykes T, Reeder C, Landau S, et al. Cognitive remediation therapy in schizophrenia: randomised controlled trial. Br J Psychiatry J Ment Sci 2007; 190: 421–7.
149. Yildiz M, Özaslan Z, Incedere A, et al. The Effect of Psychosocial Skills Training and Metacognitive Training on Social and Cognitive Functioning in Schizophrenia. Noro Psikiyatr Ars. 2018; 56(2):139-143.
150. Zhu X, Fan H, Fan F, et al. Improving social functioning in community-dwelling patients with schizophrenia: a randomized controlled computer cognitive remediation therapy trial with six months follow-up. Psychiatry Res 2020; 287:112913.
151. Zimmer M, Duncan AV, Laitano D, Ferreira EE, Belmonte-de-Abreu P. A twelve-week randomized controlled study of the cognitive-behavioral Integrated Psychological Therapy program: positive effect on the social functioning of schizophrenic patients. Rev Bras Psiquiatr Sao Paulo Braz 1999 2007; 29: 140–7.

**Ongoing studies**

1. Lopez-Morinigo J-D, Ruiz-Ruano VG, Martínez ASE, et al. Study protocol of a randomised clinical trial testing whether metacognitive training can improve insight and clinical outcomes in schizophrenia. BMC Psychiatry 2020; 20: 30.
2. Nijman SA, Veling W, Greaves-Lord K, et al. Dynamic Interactive Social Cognition Training in Virtual Reality (DiSCoVR) for social cognition and social functioning in people with a psychotic disorder: study protocol for a multicenter randomized controlled trial. BMC Psychiatry 2019; 19: 272.
3. Otto LKM, Hofstra J, Mullen MG, et al. A cognitive remediation training for young adults with psychotic disorders to support their participation in education - study protocol for a pilot randomized controlled trial. Pilot Feasibility Stud. 2020; 6:54.
